# Development and validation of a CTNNB1‐associated metabolic prognostic model for hepatocellular carcinoma

**DOI:** 10.1111/jcmm.16181

**Published:** 2020-12-09

**Authors:** Junyu Huo, Liqun Wu, Yunjin Zang

**Affiliations:** ^1^ Liver Disease Center The Affiliated Hospital of Qingdao University Qingdao China

**Keywords:** CTNNB1 mutation, hepatocellular carcinoma, metabolic, prognostic

## Abstract

Hepatocellular carcinoma (HCC) is a heterogeneous malignancy closely related to metabolic reprogramming. We investigated how CTNNB1 mutation regulates the HCC metabolic phenotype and thus affects the prognosis of HCC. We obtained the mRNA expression profiles and clinicopathological data from The Cancer Genome Atlas (TCGA), the International Cancer Genomics Consortium (ICGC) and the Gene Expression Omnibus database (GSE14520 and GSE116174). We conducted gene set enrichment analysis on HCC patients with and without mutant CTNNB1 through TCGA dataset. The Kaplan‐Meier analysis and univariate Cox regression analysis assisted in screening metabolic genes related to prognosis, and the prognosis model was constructed using the Lasso and multivariate Cox regression analysis. The prognostic model showed good prediction performance in both the training cohort (TCGA) and the validation cohorts (ICGC, GSE14520, GSE116174), and the high‐risk group presented obviously poorer overall survival compared with low‐risk group. Cox regression analysis indicated that the risk score can be used as an independent predictor for the overall survival of HCC. The immune infiltration in different risk groups was also evaluated in this study to explore underlying mechanisms. This study is also the first to describe an metabolic prognostic model associated with CTNNB1 mutations and could be implemented for determining the prognoses of individual patients in clinical practice.

## 1 INTRODUCTION

Hepatocellular carcinoma (HCC) acts as a heterogeneous disease of which the prognosis is dismal. However, recently, with the rapid development of gene sequencing technology, our understanding of the molecular pathogenesis of HCC has improved significantly.^1^, ^2^ Accumulated data from high‐throughput analysis of a large number of samples suggest that it can be used to identify key biomarkers associated with HCC progression. However, the number of biomarkers known to be associated with HCC prognosis is limited.

CTNNB1 mutations have been reported in approximately 18% to 40% of HCC patients.^3^, ^4^, ^5^, ^6^, ^7^ The oncogenic Wnt/β‐catenin pathway, activated by the mutated CTNNB1, plays a key role in the metabolic regulation in the liver.^1^, ^8^ CTNNB1‐mutated HCC has a distinctive metabolic morphotype and is often cholestatic and infrequently steatotic.^3^, ^9^, ^10^ A recent report showed that proteins involved in different metabolic activities such as drug metabolism, amino acid metabolism, glycolysis and gluconeogenesis are enriched in CTNNB1 mutant tumours,^11^ indicating that it is closely related to metabolic reprogramming. However, the potential mechanism is not fully understood.

Metabolic reprogramming refers to the significant changes in metabolic patterns that occur during the process of cell carcinogenesis, which involves many processes such as glycolysis, oxidative phosphorylation, tricarboxylic acid cycle, amino acid metabolism, nucleic acid metabolism and fatty acid metabolism.^12^, ^13^, ^14^ Tumorigenesis is a multistep process involving modifications to pathways that promote uncontrolled proliferation and eliminate cell death, which requires metabolic reprogramming to provide large molecules for cell survival, growth and migration. In recent years, targeting metabolic reprogramming has become a promising novel treating strategy, but the metabolic reprogramming of HCC has not yet been deciphered.

The study conducted gene set enrichment analysis on the CTNNB1 mutant HCC and CTNNB1 wild‐type HCC through the Cancer Genome Atlas (TCGA) database, finding that gene sets significantly up‐regulated in CTNNB1 mutant HCC were all related to metabolism, further confirming the close relationship between CTNNB1 mutation and metabolic reprogramming. We further explored the prognostic value of these genes in HCC and constructed a prognostic risk model consisting of five metabolic genes. The results of multiple datasets (including TCGA, ICGC, GSE14520 and GSE116174; total of 876 HCC patients) indicate that this risk score is highly accurate in evaluating the prognosis of HCC. In addition, we found that the prognostic model may reflect the immune microenvironment of the tumour and thus has high clinical application potential.

## 2 MATERIALS AND METHODS

### 2.1 Data collection from TCGA

We obtained the sequence data for 374 samples with HCC from the TCGA website (https://portal.gdc.cancer.gov/repository). The corresponding clinical data including overall survival time, survival status, sex, age, race, alpha‐fetoprotein (AFP) level, body mass index (BMI), vascular invasion, histological grading, AJCC TNM stage, family history of cancer, tumour history, presence of new tumours after initial treatment and individual tumour status of the TCGA cohort were obtained from the UCSC Xena website (https://xenabrowser.net/). The CTNNB1 mutation sample list was obtained from the cBioPortal website (https://www.cbioportal.org/). We retained genes of which the average expression value is over 1 and meanwhile removed RNA‐sequencing data with low abundance.^15^, ^16^ This study meets the publication requirement of TCGA (http://cancergenome.nih.gov/publications/publicationguidelines).

### 2.2 Data collection from ICGC and GEO

Three independent cohorts were used for external validation (ICGC‐LIRI‐JP, GSE14520 and GSE116174). We obtained the gene expression files (ICGC‐LIRI‐JP gene expression files from the Illumina HiSeq RNA‐seq platform, GSE14520 gene expression files from the GPL571 platform and GSE116174 gene expression files from the GPL13158 platform) and corresponding clinical data from the Gene Expression Omnibus (GEO) database (https:// www.ncbi.nlm.nih.gov/geo/ ) and the International Cancer Genomics Consortium (ICGC, https://icgc.org/). The batch effects of RNA‐seq data sets were eliminated via the combat function contained in the SVA R package (R Core Team, R Foundation for Statistical Computing, Vienna, Austria). The downloaded profiles all meet the GEO and ICGC data access rules.

### 2.3 Gene set enrichment analysis (GSEA)

Gene set enrichment analysis was utilized in this study to uncovering the differences in the metabolic‐related genes between the CTNNB1 mutant group (n = 98) and the non‐mutant group (n = 276) in the TCGA cohort. An annotated gene set file (c2.cp.kegg.v7.0.symbols.gmt) was selected as the reference. The threshold was confirmed as NOM *P*‐value < 0.05.

### 2.4 Construction and validation of a metabolic‐related prognostic model

We first used univariate Cox regression and the Kaplan‐Meier (K‐M) for survival analysis of each gene and selected genes with *P* < 0.05 in both algorithms as candidate genes to construct the model.^17^ Next, the Lasso regression analysis assisted in further narrowing down the candidate gene number. Finally, we constructed a risk score system, multiplying the normalized expression level exhibited by each metabolic gene by virtue of the regression coefficients that were obtained from the multivariate Cox regression analysis. The median risk score of the TCGA cohort (n = 343) was used to classify the group with a high risk and group with a low risk. The Kaplan‐Meier (K‐M) survival analysis (log‐rank test), together with the time‐related receiver operating feature curve (ROC) analysis applied assessing the predictive ability exhibited by the prognostic model in above‐mentioned four independent cohorts (TCGA, ICGC, GSE14520 and GSE116174). Univariate and multivariate Cox regression analyses were performed in this study to confirm whether the risk score could independently predict the prognosis. *P* < 0.05 was considered with statistical significance. K‐M method in the R package ‘survminer’ was used to generate the survival curves, and R package ‘survivalROC’ was used to generate ROC curves. What needs to be pointed out is that in order to avoid the impact of other factors on the prognosis of patients, we excluded patients with a survival time of less than one month.

### 2.5 Correlation analysis between clinicopathological parameters and risk score

We used the Wilcoxon signed‐rank test (2 groups) or the Kruskal‐Wallis test (>=2 groups) for analysing the correlation between clinicopathology (including AJCC TNM stage, Barcelona stage, CLIP stage, main tumour size, tumour differentiation, AFP and vascular tumour cell type) and the risk score. *P* < 0.05 was considered with statistical significance, and box plots were generated via the beeswarm packages implemented in R software.

### 2.6 Identification of differentially expressed genes (DEGs) between different risk groups

DEGs in the tissues of the two HCC groups were examined using the Wilcoxon test method in R package ‘limma’. The thresholds were confirmed to be |log2‐fold change (FC)| > 1.0 and FDR < 0.05. Gene ontology (GO) enrichment analysis was further used for biological function annotation of the DEGs using the R package ‘clusterProfiler’.

### 2.7 Evaluation of immune cell infiltration in different risk groups

Relevant data about the infiltration level exhibited by immune cells of HCC patients were downloaded from the tumour immune assessment resource (TIMER) website to compare the immune cell infiltration level between different risk groups in the TCGA dataset. The single sample gene set enrichment analysis (ssGSEA, the immune cell type, function and pathway represented by 29 immune‐related gene sets) was employed to quantify the activity or enrichment levels of immune cells, functions or pathways in the high‐ and low‐risk samples from the four independent cohorts. The normalized enrichment score (NES) that calculated from the ssGSEA was used in the ‘GSVA’ and ‘GSEABase’ R package. The independent‐samples t tests were used for comparing the differences in immune infiltration levels and immune function between high‐ and low‐risk groups, and *P*‐values < 0.05 were suggested to exhibit statistical significance.

### 2.8 Statistical analyses

R software v3.6.1 (R Foundation for Statistical Computing, Vienna, Austria) together with GraphPad Prism v7.00 (GraphPad Software Inc, USA) assisted in performing statistical analyses. Fisher's exact test or Pearson's chi‐square test helped to analyse qualitative variables. Analysis on quantitative variables relied on a non‐parametric Wilcoxon rank‐sum test (for unpaired samples). The Kruskal‐Wallis test was applied for normalizing multiple groups. If not specified above, *P* < 0.05 was considered with statistical significance.

## 3 RESULTS

### 3.1 Identification of differential metabolic gene sets between HCC samples with and without CTNNB1 mutations

**Figure 1:**
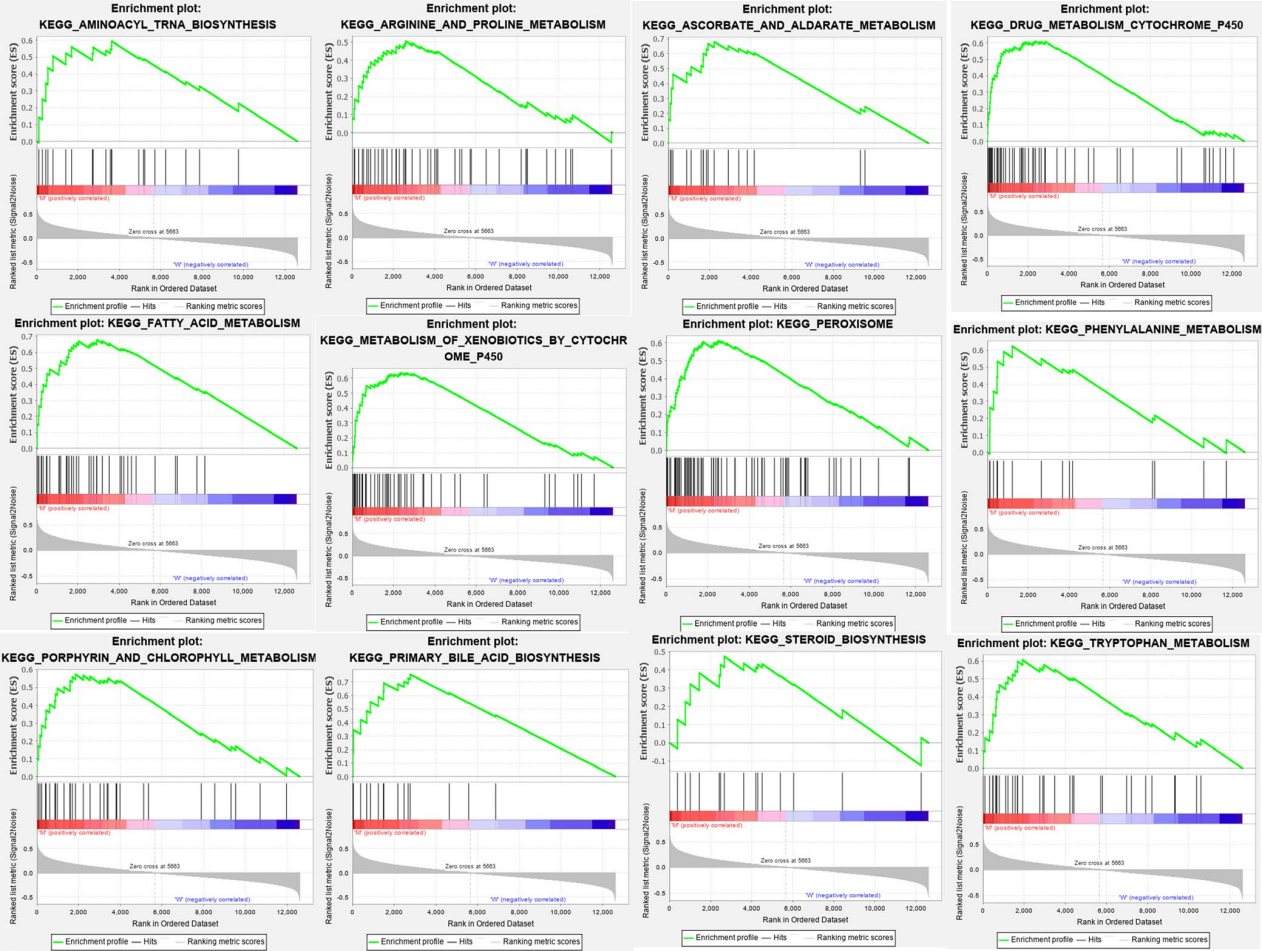
Gene set enrichment analysis between with and without CTNNB1 mutation HCC in TCGA

Although CTNNB1 mutant HCC has been reported to have unique metabolic characteristics, the associated metabolic genes are still unknown.^3^ GSEA showed that the gene sets significantly up‐regulated in CTNNB1 mutant HCCs were all associated with metabolic pathways (NOM *P* < 0.05) (Figure 1). These included ‘metabolism of xenobiotics by cytochrome P450’ (NES = 1.914, NOM *P* = 0.002), ‘drug metabolism—cytochrome P450’ (NES = 1.813, NOM *P* = 0.006), ‘peroxisome’ (NES = 1.767, NOM *P* = 0.02), ‘tyrosine metabolism’ (NES = 1.758, NOM *P* = 0.01), ‘porphyrin and chlorophyll metabolism’ (NES = 1.745, NOM *P* = 0.002), ‘steroid hormone biosynthesis’ (NES = 1.725, NOM *P* = 0.015), ‘primary bile acid biosynthesis’ (NES = 1.710, NOM *P* = 0.01), ‘aminoacyl‐tRNA biosynthesis’ (NES = 1.707, NOM *P* = 0.02), ‘fatty acid metabolism’ (NES = 1.707, NOM *P* = 0.04), ‘ascorbate and aldarate metabolism’ (NES = 1.700, NOM *P* = 0.01), ‘phenylalanine metabolism’ (NES = 1.62, NOM *P* = 0.02) and ‘arginine and proline metabolism’ (NES = 162, NOM *P* = 0.04) (Table 1). In contrast, no gene sets related to metabolic pathways were significantly enriched in wild‐type CTNNB1 HCC.

**Table 1:** Gene set enrichment analysis between with and without CTNNB1 mutant HCC

NAME	NES	NOM *P*‐value
KEGG_METABOLISM_OF_XENOBIOTICS_BY_CYTOCHROME_P450	1.914358	0.002132
KEGG_DRUG_METABOLISM_CYTOCHROME_P450	1.81373	0.006383
KEGG_PEROXISOME	1.767487	0.02439
KEGG_TYROSINE_METABOLISM	1.758547	0.012876
KEGG_PORPHYRIN_AND_CHLOROPHYLL_METABOLISM	1.745985	0.026477
KEGG_STEROID_HORMONE_BIOSYNTHESIS	1.725979	0.015317
KEGG_PRIMARY_BILE_ACID_BIOSYNTHESIS	1.710733	0.010331
KEGG_AMINOACYL_TRNA_BIOSYNTHESIS	1.707652	0.025052
KEGG_FATTY_ACID_METABOLISM	1.707306	0.046939
KEGG_ASCORBATE_AND_ALDARATE_METABOLISM	1.700901	0.014737
KEGG_PHENYLALANINE_METABOLISM	1.627591	0.028509

### 3.2 Construction of prognostic model in the TCGA cohort

**Figure 2:**
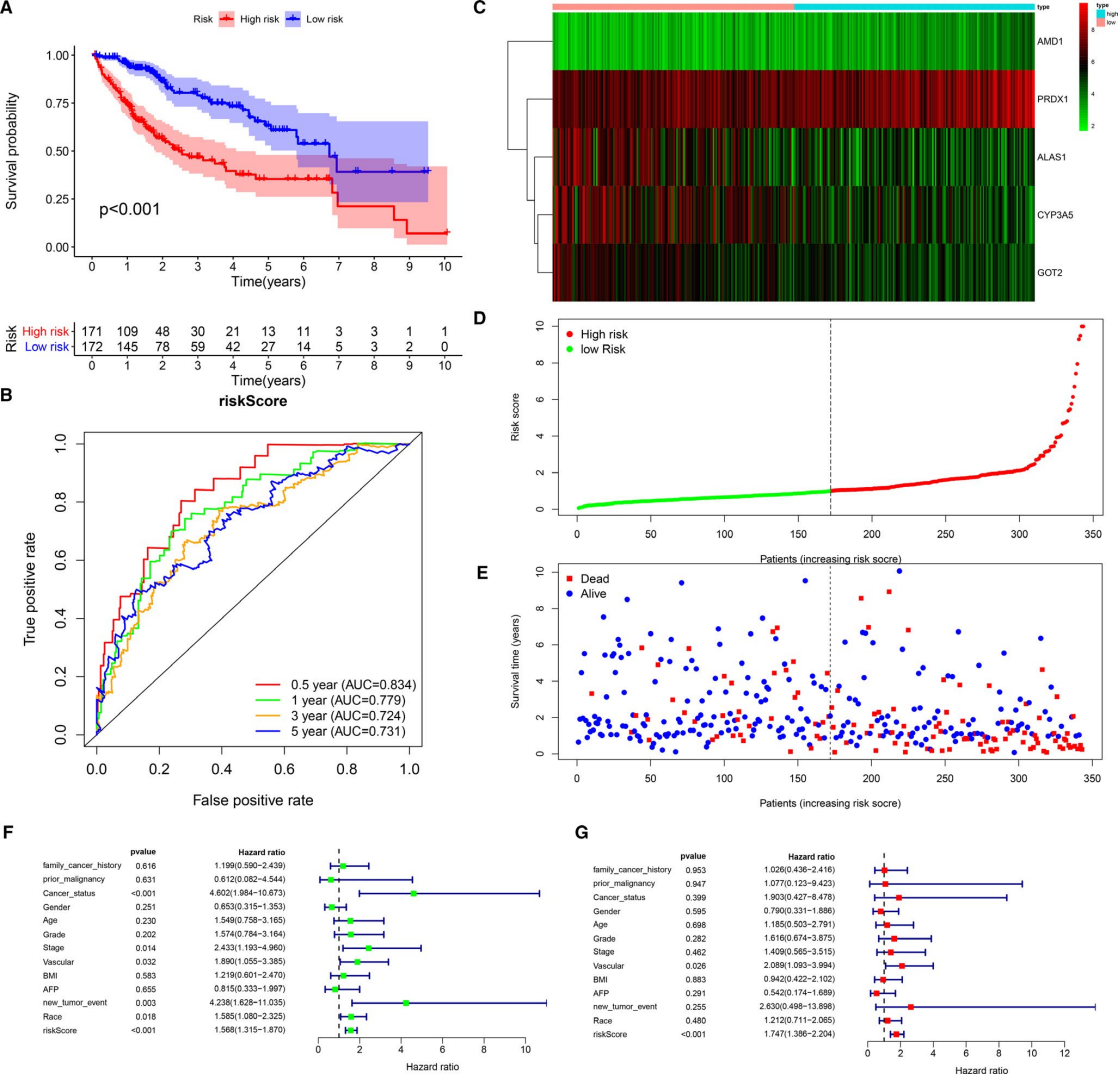
Construction of the prognostic model in TCGA cohort (A) The Kaplan‐Meier survival analysis for overall survival (OS) of patients in TCGA cohort. (B) The time‐dependent ROC analysis for risk score in the TCGA cohort. (C‐E) The heat map of the five genes and the distribution of risk score and the survival status of patients in the TCGA cohort. (F‐G) Forest plot of the univariate and multivariate Cox regression analysis in HCC regarding OS (green represents univariate analysis, and red represents multivariate analysis)

Although CTNNB1 has been confirmed to be one of the genes with the highest mutation frequency in HCC,^1^, ^2^, ^3^, ^4^, ^7^, ^18^ whether the significantly enriched metabolic gene set in CTNNB1 mutant HCC affects the prognosis of HCC is still unclear. Through univariate Cox regression analysis and K‐M survival analysis, we identified 14 genes most associated with overall survival of HCC (Table 2). We used the Lasso regression and multi–Cox regression to further narrow down the range of model genes to optimize the model. Finally, a risk score consisting of five metabolic genes was constructed: risk score (RS) = normalized expression level of CYP3A5 * −0.00248 + normalized expression level of ALAS1 * −0.00445 + normalized expression level of PRDX1 * 0.003267 + normalized expression level of GOT2 * −0.00887 + normalized expression level of AMD1 * 0.078805. The median risk score of the TCGA cohort was used as the unified cut‐off for dividing the group with a high risk (RS > 1) and the group with a low risk (RS < 1). The group with a high risk presented an obviously lower OS relative to the group with a low risk (Figure 2A), and the area under the ROC curve of the prognosis model at 0.5, 1, 3 and 5 years was 0.834, 0.779, 0.724 and 0.731, respectively (Figure 2B). The expression levels of ALAS1, CYP3A5 and GOT2 were higher in the low‐risk group, whereas the expression levels of AMD1 and PRDX1 were higher in the high‐risk group (Figure 2C). The risk of death in HCC patients increased with the increase in risk score (Figure 2D‐E). As found by the univariate and multivariate Cox regression analyses, the risk score could independently predict the prognosis (Figure 2F‐G).

**Table 2:** The prognostic gene list related to overall survival in TCGA cohort

Gene	K‐M	HR	HR.95L	HR.95H	Cox *P*‐value
CYP27A1	0.004001	0.995377	0.992811	0.99795	0.000435
AGPS	0.002754	1.14859	1.083584	1.217495	3.15E‐06
CYP3A5	0.000394	0.994496	0.991012	0.997993	0.002057
HMBS	0.005014	1.085504	1.021233	1.15382	0.008422
ADH1A	0.001722	0.997199	0.995436	0.998966	0.001897
SRM	0.00237	1.015787	1.00748	1.024162	0.000185
ACSL3	0.002632	1.047003	1.024575	1.069922	3.22E‐05
ALAS1	0.009778	0.992547	0.988309	0.996803	0.000611
PRDX1	0.002291	1.004368	1.002789	1.00595	5.63E‐08
GOT2	0.003586	0.983121	0.974783	0.99153	8.95E‐05
HCCS	0.000666	1.113809	1.047345	1.184489	0.000596
AMD1	4.61E‐06	1.110299	1.068779	1.153433	7.42E‐08
CYP2C9	0.00024	0.99541	0.992975	0.997852	0.000233
SMS	3.01E‐06	1.035489	1.022567	1.048574	5.23E‐08

### 3.3 Internal validation of the prognostic model in TCGA cohort

**Figure 3:**
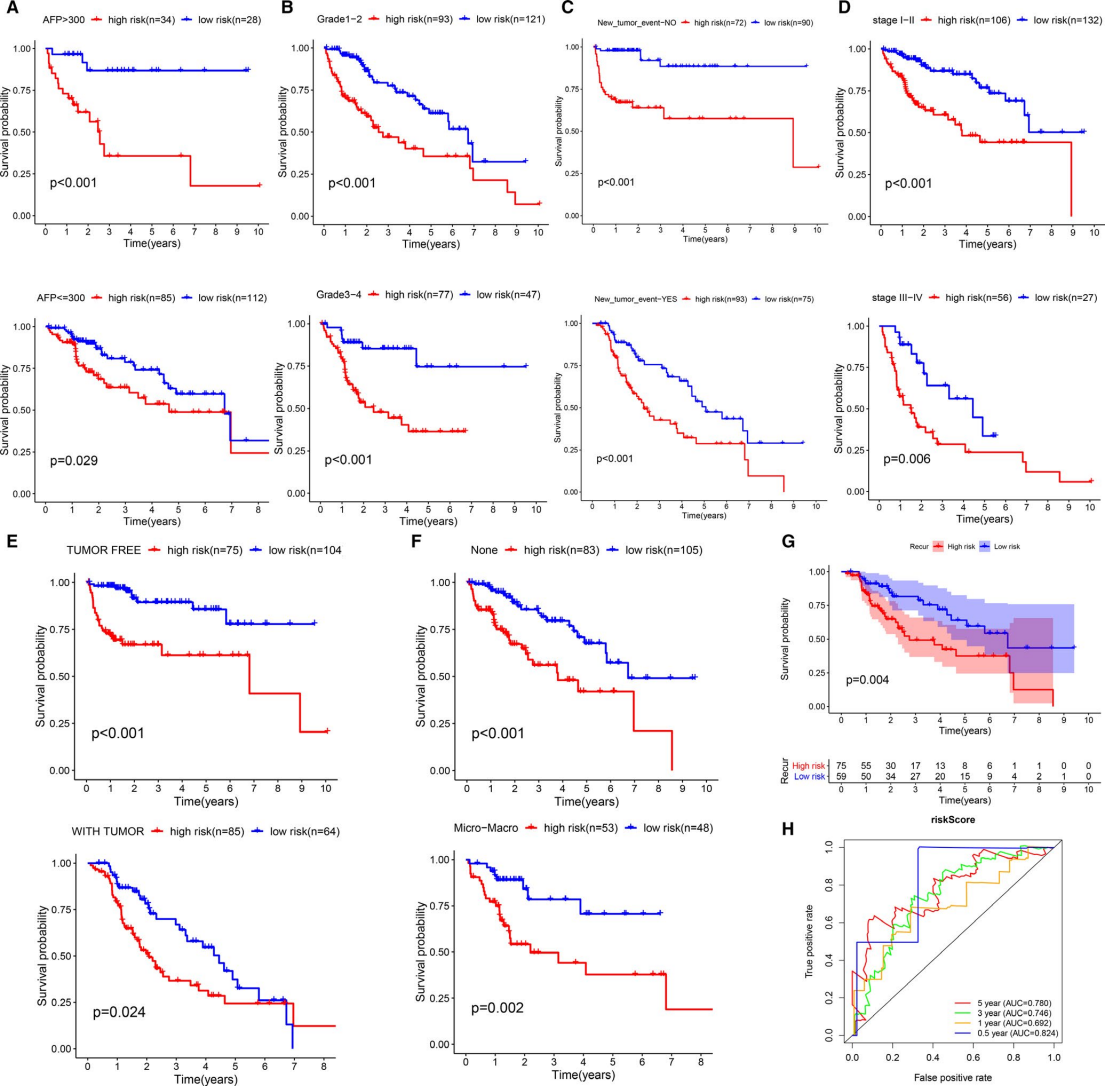
Internal validation of the prognostic model in TCGA cohort based on clinical features (A) AFP. (B) Histopathological grade. (C) New tumour event after initiate treatment. (D) AJCC TNM stage. (E) Cancer status. (F) Vascular tumour cell type. (G‐H) Recurrence

Patients fell into 12 subgroups for survival analysis according to their clinical characteristics such as AFP levels, vascular invasion, histological grade, AJCC TNM stage, new tumour after initial treatment and individual tumour status. In each subgroup, the OS rate of the group with a high risk appeared lower relative to the group with a low risk (Figure 3A‐F). Also, the prognostic model accurately evaluated the prognosis of 134 patients with recurrence (including 65 cases of intrahepatic recurrence, 46 cases of local recurrence and 23 cases of extrahepatic recurrence). The area under the ROC curve at 0.5, 1, 3 and 5 years was 0.824, 0.692, 0.746 and 0.780, respectively (Figure 3H).

### 3.4 External validation of the prognostic model in three different independent cohort

**Figure 4:**
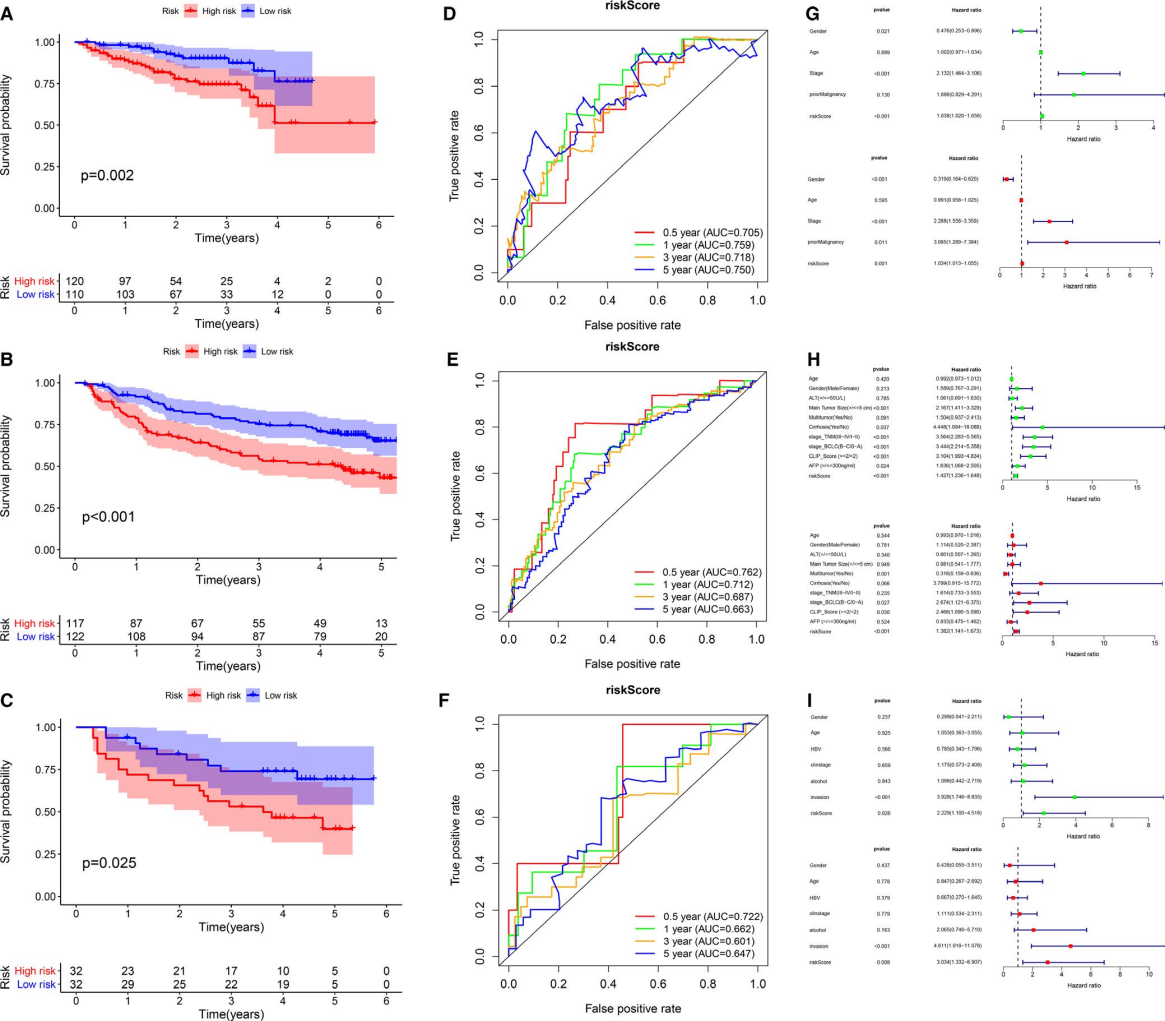
External validation of the prognostic model in three independent cohort (A‐C) The Kaplan‐Meier curve of overall survival (OS) in ICGC cohort, GSE14520 cohort and GSE116174 cohort, respectively. (D‐F) The time‐dependent ROC analysis for risk score in ICGC cohort, GSE14520 cohort and GSE116174 cohort, respectively. (G‐I) Forest plot of the univariate and multivariate Cox regression analysis regarding OS in ICGC cohort, GSE1452 cohort and GSE116174 cohort, respectively (green represents univariate analysis, and red represents multivariate analysis)

For verifying whether the prognostic model is robust, we used three independent cohorts (ICGC (n = 230), GSE14520 (n = 239) and GSE116174 (n = 64)) for external validation. Using the same formula and cut‐off obtained from the TCGA‐HCC cohort, patients were assigned to the group with a high risk and group with a low risk. Consistent with the results of TCGA, the group with a high risk showed significantly poorer OS relative to the group with a low risk (Figure 4A‐C). In the ICGC cohort, the areas under the ROC curve at 0.5, 1, 3 and 5 years were 0.705, 0.759, 0.718 and 0.750, respectively (Figure 4D). In the GSE14520 cohort, the area under the ROC curve at 0.5, 1, 3 and 5 years was 0.762, 0.712, 0.687 and 0.663, respectively (Figure 4E). In the GSE116174 cohort, the areas under the ROC curve at 0.5, 1, 3 and 5 years were 0.722, 0.662, 0.601 and 0.647, respectively (Figure 4F). As demonstrated in the univariate and multivariate Cox regression analysis, the risk score could independently predict the prognosis in each cohort (Figure 4G‐I). These results indicate that the prognostic model we constructed was capable of general application.

### 3.5 Relationship between risk score and clinicopathological characteristics

**Figure 5:**
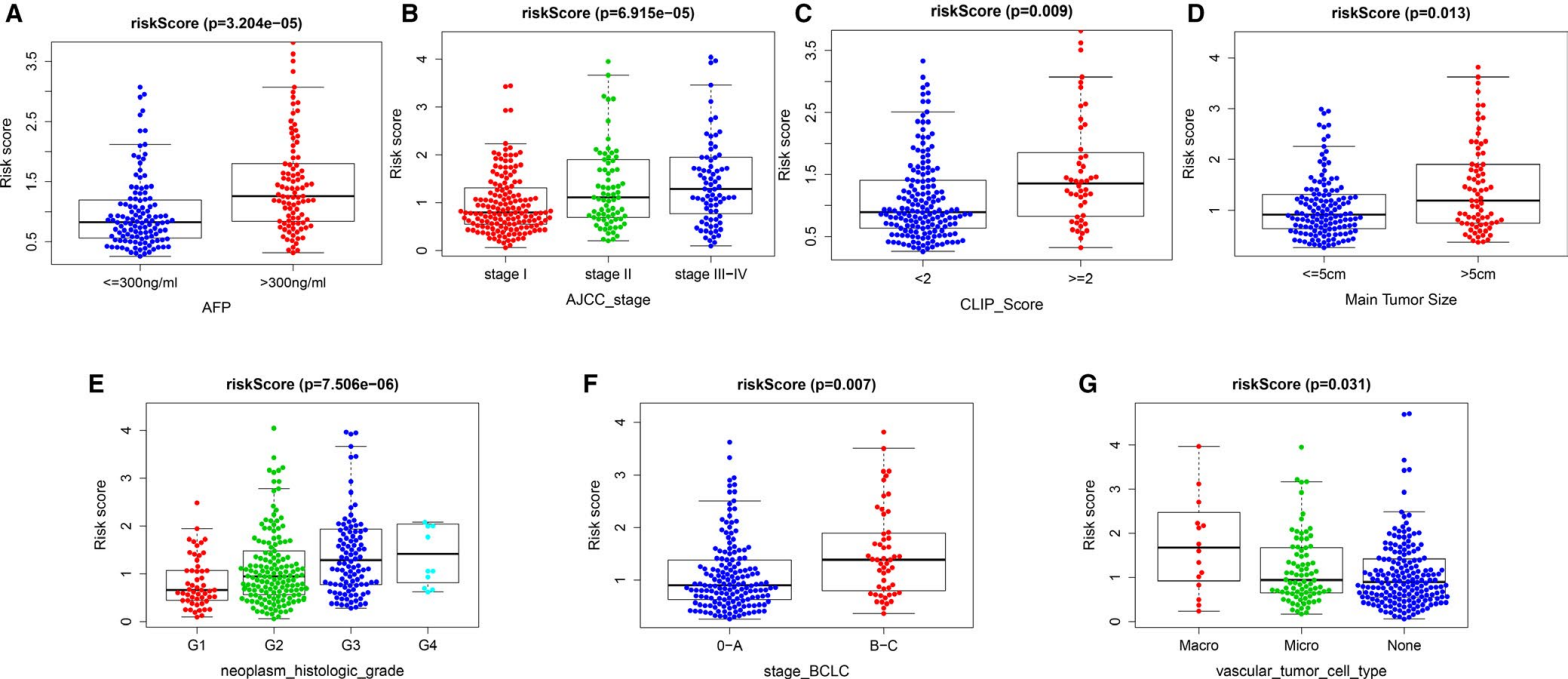
Correlation analysis between risk score and clinicopathological characteristics (A) AFP. B, AJCC stage. C, CLIP stage. D, Main tumour size. E, Histopathological grade. F, BCLC stage. G, Vascular tumour cell type

Previous studies have shown that AFP levels, histological grade, clinical stage (TNM, BCLC and CLIP), tumour diameter and vascular invasion are correlated with the prognosis of HCC.^19^, ^20^, ^21^, ^22^ The present study analysed the correlation between risk score and the prognostic factors by making use of the clinical data of the four independent cohorts. Results showed that higher‐risk scores were significantly associated with higher AFP levels, higher histological grade, larger tumour diameter, vascular invasion and advanced clinical staging (TNM, BCLC and CLIP) (Figure 5). We also performed the chi‐square tests on different risk groups for correlation analysis of clinical features. The results showed that the two groups were remarkably different regarding the clinical stage, histological grade, number of tumours, AFP levels and survival status (Tables 3, 4, 5, 6).

**Table 3:** The chi‐square test of the relation between risk score and clinical features in TCGA cohort

Clinical features	Risk score		χ^2^	*P*‐value
	Low risk n (%)	High risk n (%)		
AFP				
>300 ng/mL	28 (20.00%)	34 (28.57%)	2.596	0.1072
≤300 ng/mL	112 (80.00%)	85 (71.43%)		
Age				
>65	64 (37.21%)	63 (36.84%)	0.004959	0.9439
≤65	108 (62.79%)	108 (63.16%)		
Gender				
Female	51 (29.65%)	59 (34.50%)	0.9266	0.3358
Male	121 (70.35%)	112 (65.50%)		
BMI				
>25	82 (51.25%)	71 (45.51%)	1.041	0.3076
≤25	78 (48.75%)	85 (54.49%)		
Histological grade				
G1‐2	121 (72.02%)	93 (54.71%)	10.91	0.001
G3‐4	47 (27.98%)	77 (45.29%)		
New tumour event after initiate treatment				
Yes	75 (45.45%)	93 (56.36%)	3.929	0.0475
No	90 (54.55%)	72 (43.64%)		
Prior malignancy				
Yes	18 (10.47%)	13 (7.60%)	0.8548	0.3552
No	154 (89.53%)	158 (92.40%)		
Race				
White	80 (50.63%)	89 (55.97%)	0.9086	0.3405
Asian	78 (49.37%)	70 (44.03%)		
Family cancer history				
Yes	44 (38.60%)	29 (35.37%)	0.213	0.6444
No	70 (61.40%)	53 (64.63%)		
AJCC TNM stage				
I‐II	132 (83.02%)	106 (65.43%)	12.95	0.0003
III‐IV	27 (16.98%)	56 (34.57%)		
Cancer status				
With tumour	64 (38.10%)	85 (53.13%)	7.467	0.0063
Tumour free	104 (61.90%)	75 (46.88%)		
Vascular invasion				
None	105 (68.63%)	83 (61.03%)	1.828	0.1763
Yes	48 (31.37%)	53 (38.97%)		
Survival status				
Alive	90 (70.31%)	134 (62.33%)	2.259	0.1328
Dead	38 (29.69%)	81 (37.67%)		

**Table 4:** The chi‐square test of the relation between risk score and clinical features in ICGC cohort

Clinical feature	Risk score		χ^2^	*P*‐value
	High risk n (%)	Low risk n (%)		
Survival status				
Alive	91 (48.15%)	98 (51.85%)	6.886	0.009
Dead	29 (70.73%)	12 (29.27%)		
Gender				
Male	85 (50.30%)	84 (49.70%)	0.901	0.343
Female	35 (57.38%)	26 (42.62%)		
Age				
>65	75 (53.19%)	66 (46.81%)	0.151	0.698
≤65	45 (50.56%)	44 (49.43%)		
Prior malignancy				
Yes	18 (60%)	12 (40%)	0.847	0.357
No	102 (51%)	98 (49%)		
Stage				
I*‐*II	66 (46.48%)	76 (53.52%)	4.824	0.028
III‐IV	54 (61.36%)	34 (38.64%)		

**Table 5:** The chi‐square test of the relation between risk score and clinical features in GSE14520 cohort

Clinical feature	Risk score		χ^2^	*P*‐value
	High risk n (%)	Low risk n (%)		
Survival status				
Alive	54 (46.24%)	78 (53.76%)	8.5	0.004
Dead	52 (49.59%)	33 (50.41%)		
Gender				
Male	91 (50.30%)	98 (49.70%)	0.287	0.592
Female	15 (57.38%)	13 (42.62%)		
Age				
>65	4 (53.19%)	15 (46.81%)	7.715	0.005
≤65	102 (50.56%)	96 (49.43%)		
ALT				
>50 U/L	50 (56.18%)	39 (53.82%)	3.471	0.062
≤50 U/L	55 (43.31%)	72 (56.69%)		
AFP				
>300 ng/mL	65 (67.01%)	32 (32.99%)	23.157	<0.001
≤300 ng/mL	41 (34.17%)	79 (65.83%)		
Stage_TNM				
I‐II	72 (46.48%)	96 (53.52%)	10.686	0.001
III‐IV	34 (61.36%)	15 (38.64%)		
Main tumour size				
>5 cm	44 (57.14%)	33 (42.86%)	3.487	0.062
≤5 cm	61 (43.88%)	78 (56.12%)		
Multitumour				
Solitary	75 (44.38%)	94 (55.62%)	5.569	0.018
Multiple	30 (63.83%)	17 (36.17%)		
Cirrhosis				
Yes	100 (50.25%)	99 (49.75%)	2.723	0.099
No	5 (29.41%)	12 (70.59%)		
Stage_BCLC				
0‐A	70 (42.68%)	94 (57.32%)	9.584	0.002
B‐C	35 (67.31%)	17 (32.69%)		
CLIP_Score				
≥2	35 (72.92%)	13 (27.08%)	14.595	<0.01
<2	70 (41.67%)	98 (58.33%)		

**Table 6:** The chi‐square test of the relation between risk score and clinical features in GSE116174 cohort

Clinical feature	Risk score		χ^2^	*P*‐value
	High risk n (%)	Low risk n (%)		
Survival status				
Alive	14 (37.84%)	23 (62.16%)	5.189	0.023
Dead	18 (66.67%)	9 (33.33%)		
Gender				
Male	3 (50%)	3 (50%)	0.184	0.668
Female	29 (50%)	29 (50%)		
Age				
>65	3 (33.33%)	6 (66.67%)	2.283	0.131
≤65	29 (52.73%)	26 (47.27%)		
HBV				
+	21 (44.68%)	26 (55.32%)	2.003	0.157
−	11 (64.71%)	6 (35.29%)		
Clinical stage				
I‐II	24 (45.28%)	29 (54.72%)	3.952	0.047
III	8 (72.73%)	3 (27.27%)		
Alcohol				
Yes	4 (30.77%)	9 (69.23%)	3.475	0.062
No	28 (54.90%)	23 (45.10%)		
Invasion				
Yes	14 (48.28%)	15 (51.72%)	0.063	0.802
No	18 (51.43%)	17 (48.57%)		

### 3.6 Gene Ontology functional enrichment analysis

**Figure 6:**
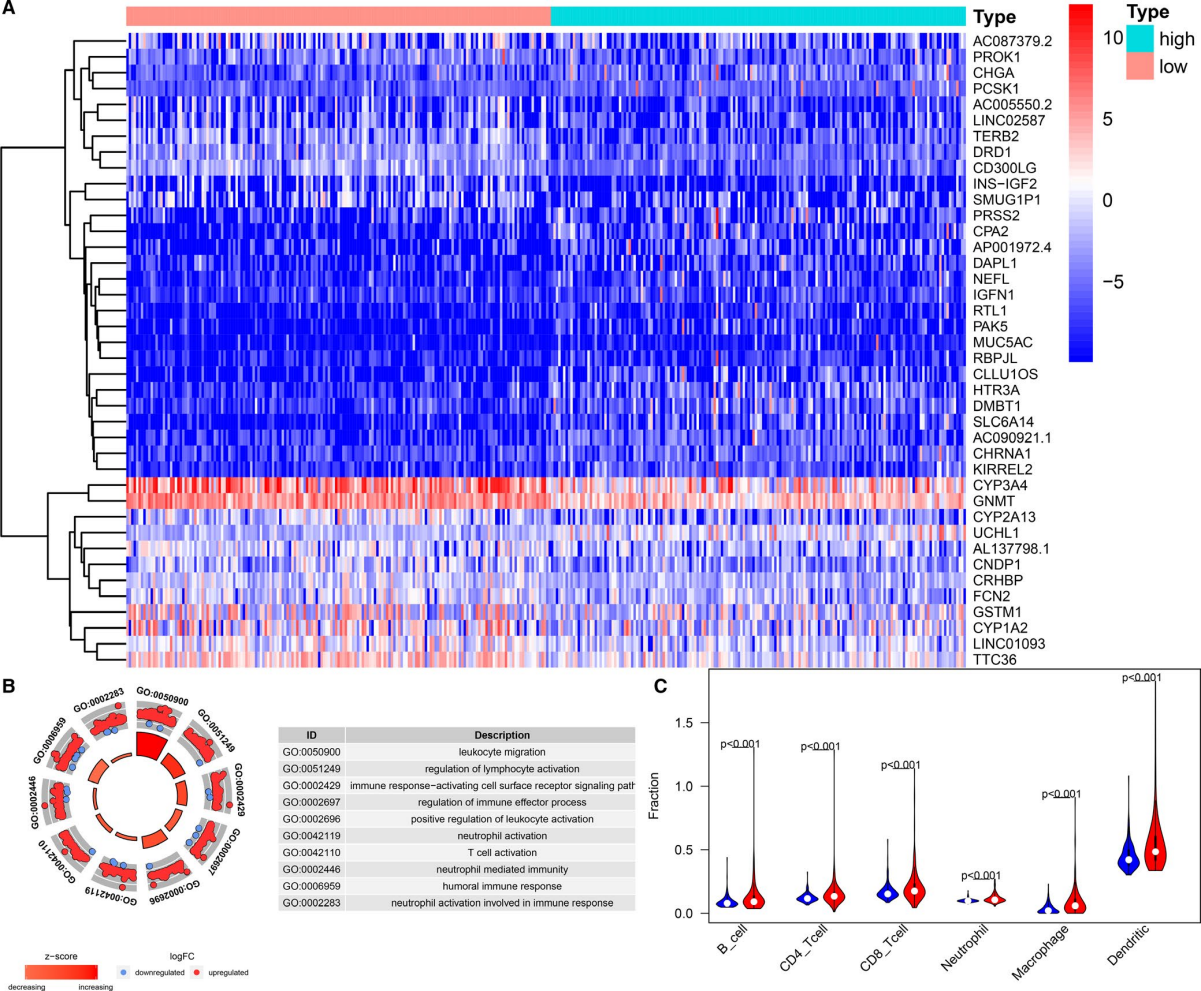
Identification of differentially expressed genes in high‐risk and low‐risk HCC patients. A, Heat map of differentially expressed genes samples between high‐risk and low‐risk groups. B, GO circle plot of immune pathways differentially enriched in patients with different risk scores. C, Violin plot of relationships between the risk score and infiltration abundances of six types of immune cells (red represents high‐risk group, and blue represents low‐risk group)

We used Gene Ontology (GO) functional enrichment analysis to annotate the function of DEGs (Figure 6A) in the group with a high risk and the group with a low risk. As found, the genes significantly up‐regulated in the group with a high risk were associated with a variety of immune regulatory processes including regulation of lymphocyte activation, regulation of immune effector processes and humoral immune response (Figure 6B). The estimation of immune cell infiltration used by TIMER method showed that the infiltration level of six types of immune cells in high‐risk group was all higher than that in low‐risk group (Figure 6C), indicating that the prognostic signature may affect the prognosis of HCC patients through regulating immune microenvironment of tumour.

### 3.7 Relationship between risk score and immune cell infiltration

**Figure 7:**
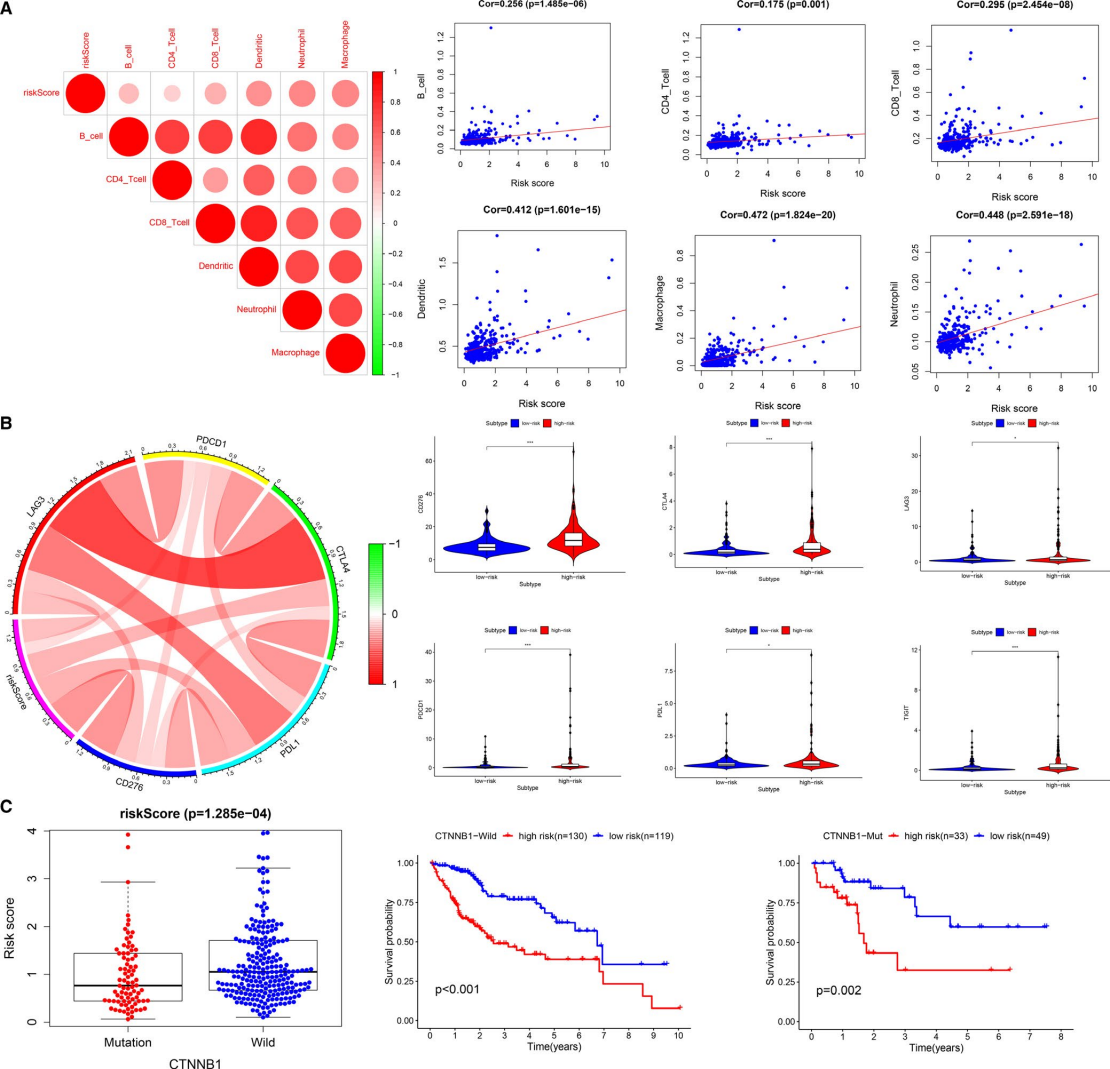
The landscape of immune infiltration in high‐ and low‐risk HCC patients. A, Correlation analysis of risk score and immune cell infiltration. B, The relationship between risk score and expression level of immune checkpoint. C, The relationship between risk score and CTNNB1 status (*P*‐value significant codes: 0 ≤ *** < 0.001 ≤ ** < 0.01 ≤ * < 0.05 ≤ . < 0.1)

We further analysed the correlation between the prognostic model and immune cells’ infiltration, and found that the risk score exhibited a positive relation to the infiltration of six types of immune cells: CD4 T cells (*r* = 0.175, *P* = 0.001), CD8 T cells (*r* = 0.295, *P* < 0.001), B cells (*r* = 0.256, *P* < 0.001), macrophages (*r* = 0.472, *P* < 0.001), dendritic cells (*r* = 0.412, *P* < 0.001) and neutrophils (*r* = 0.448, *P* < 0.001) (Figure 7A). CTNNB1‐mutated HCC is characterized by immune rejection,^23^ and a recent clinical study showed that this subgroup is not sensitive to the treatment of immune‐checkpoint inhibitors.^8^ Our study found that the risk score positively corrected with the expression levels of six major immune checkpoints (PDL1, LAG3, CTLA4, CD276, PDCD1 and TIGIT) (Figure 7B), which were consistent with the risk score of the CTNNB1‐mutated subgroup, which was lower than that of the non‐mutated subgroup (Figure 7C).

### 3.8 Relationship between risk score and tumour immune microenvironment

We used ssGSEA to further explore the internal relationship between prognostic model and tumour immune microenvironment (TIME). In terms of immune cell infiltration, the abundance of Tregs, aDCs, Th2 cells and macrophages of the high‐risk group was significantly higher than those of the low‐risk group in the four independent cohorts (Figure S1A‐D). In terms of immune function, the results revealed that the type II IFN response in the low‐risk group was significantly stronger than that in the low‐risk group (Figure S1A‐D). In addition, the expression of immune checkpoint‐related gene set was up‐regulated in the high‐risk group, which was consistent with the previous results (Figure S1A‐B).

## 4 DISCUSSION

In the past few decades, considerable progress has been made in understanding the risk factors and molecular characteristics of HCC.^24^, ^25^, ^26^ However, morbidity and cancer‐specific mortality continue to increase in many countries.^26^, ^27^ The existing prognostic staging system still has many limitations in guiding accurate treatment and predicting more accurate clinical outcome.^28^, ^29^ We need to further improve the existing prognostic staging system using gene sequencing technology, so that patients can benefit from the accurate treatment.^8^ There is increasing evidence that metabolic reprogramming can remarkably affect HCC development and may be related to advanced diseases and adverse clinical outcome^30^, ^31^; hence, it seems that targeting tumour metabolism can promisingly assist in effectively treating HCC.

A recent report found that proteins involved in different metabolic activities such as drug metabolism, gluconeogenesis, glycolysis and amino acid metabolism are enriched in CTNNB1 mutant HCC.^11^ NRF2 mutation is an early event in the carcinogenesis of HCC and is considered to be an important factor in promoting the progression of precancerous hepatocytes to HCC.^32^ Existing studies have found that the abnormal transduction of NRF2 signal pathway is associated with simvastatin.^33^, ^34^ In contrast, CTNNB1 mutation usually occurs in the later stage of HCC progression, and the relevant research on its specific mechanism is still lacking. Considering this, we conducted GSEA of mRNA expression profiles in the TCGA database. According to whether CTNNB1 was mutated, 374 tumour tissues were divided into two groups, among which 98 were mutated and 276 were not mutated. Interestingly, the significantly enriched genes in the mutant group were associated with 12 metabolic pathways. We used univariate Cox regression, K‐M survival analysis, Lasso regression and multi–Cox regression to analyse metabolic genes step by step, and ultimately constructed a five‐metabolic gene (including AMD1, PRDX1, ALAS1, CYP3A5 and GOT2) risk score model. The unified risk score formula and the threshold value of risk classification were taken into account for dividing all included patients in group with a high risk and in group with a low risk. We first conducted internal validation of the prognostic model based on the clinical characteristics of the TCGA cohort such as the presence of a new tumour after initial treatment, individual tumour status, AJCC stage, histological grade, vascular invasion and AFP levels. The patients were then divided into 12 subgroups. Based on the K‐M survival curve, in each subgroup, the group with a high risk exhibited an obviously lower OS relative to the group with a low risk. Subsequently, we conducted external verification of the prognostic model by using three independent queues, namely ICGC (n = 230), GSE14520 (n = 239) and GSE116174 (n = 64). Consistent with the TCGA results, the group with a high risk showed an obviously worse prognosis relative to the group with a low risk. As demonstrated by the univariate and multivariate Cox regression analysis, this prognostic model could independently predict the prognosis of HCC. These results support that our prognostic model has a strong general applicability. GO enrichment analysis showed that genes with up‐regulated expression in the group with a high risk showed a close relation to the immune regulation. The correlation between tumours with infiltrating macrophages and risk score was the most prominent. It is well known that macrophages are the most abundant in tumour tissues and significantly regulate tumours, which is be capable of promoting tumour cells in terms of the proliferation, invasion and metastasis as well as inducing immune tolerance in these cells.^35^, ^36^, ^37^ As expected, the risk score presented a positive relation to the expression levels of six commonly used immune checkpoints (PDL1, LAG3, CTLA4, CD276, PDCD1 and TIGIT). We conclude that the immunosuppressive tumour microenvironment may be a major factor contributing to poor prognosis in the group with a high risk. It is noteworthy that the risk score is most strongly associated with the emerging immune checkpoint CD276 (B7‐H3), which has recently been reported to be highly overexpressed in a variety of human solid cancers and is often associated with adverse clinical outcome in patients, making it a potential target for tumour immunotherapy.^38^, ^39^ Besides, high‐risk score could reflect adverse survival outcome‐related clinical characteristics, such as AFP levels (>300 ng/mL), tumour vascular invasion, low tumour differentiation, advanced AJCC stage, advanced BCLC stage, advanced CLIP stage, new tumour after initial treatment, main tumour size > 5 cm and multiple tumours, which may also help to explain the reason for the poor prognosis of high‐risk patients.

Adenosylmethionine decarboxylase 1 (AMD1) acts as an essential enzyme that affects the biosynthesis of polyamines including spermine. Zabala‐Letona found that the up‐regulation of AMD1 could activate the PTEN‐PI3K‐mTORC1 pathway to maintain the growth and proliferation of prostate cancer cells.^40^ Furthermore, Xu reported that AMD1 has tumorigenic effects on the prognosis of human gastric cancer, but the potential function of AMD1 in HCC is still unclear.^41^ Peroxiredoxin 1 (PRDX1) acts as a peroxidase family member of the antioxidant enzymes. Fang Y and Sun et al found that high expression of PRDX1 in HCC tissues corresponds to adverse clinical outcome, and the mechanism may be related to promoting tumour angiogenesis and regulating cell migration and invasion.^42^, ^43^ 5′‐Aminolevulinate synthase 1 (ALAS1) plays the role of an rate‐limiting enzyme during haem biosynthesis. As demonstrated by studies performed recently, ALAS1 can affect many cellular functions and has important effects on non–small cell lung cancer, colorectal cancer and oral cancer.^44^, ^45^ However, the role of ALAS1 in HCC remains not well known. CYP3A5 is a cytochrome P450 protein that plays a role in the metabolism of many carcinogens and anticancer drugs in the liver. Jiang et al found that CYP3A5 plays an antitumour role in HCC by regulating the mTORC2/Akt signalling pathway.^46^ Glutamate oxaloacetate transaminase 2 (GOT2) has been repeatedly reported in recent years to be associated with the progression of pancreatic cancer,^47^, ^48^ but its role in HCC remains unclear.

Although the above studies support that our prognostic model has a high potential for clinical application, the study faces some limitations. Although the accumulated data of high‐throughput analysis from a large number of samples have been optimally applied, further verification through prospective studies is necessary. Furthermore, the specific biological functions of the five genes in HCC need to be explored experimentally.

## 5 CONCLUSIONS

We constructed a novel prognostic model that is useful for further improving the prognostic evaluation system of patients with HCC, and the five metabolic genes in this model are expected to become potential targets for the treatment of HCC.

## CONFLICT OF INTEREST

The authors have no conflicts of interest to declare.

## AUTHOR CONTRIBUTION

**Junyu Huo:** Conceptualization (equal); Data curation (equal); Formal analysis (lead); Investigation (lead); Methodology (lead); Resources (lead); Software (lead); Supervision (equal); Validation (equal); Visualization (lead); Writing‐original draft (lead); Writing‐review & editing (equal). **Liqun Wu:** Conceptualization (equal); Project administration (equal); Supervision (equal); Validation (equal); Writing‐review & editing (equal). **Yunjin Zang:** Conceptualization (supporting); Data curation (supporting); Project administration (supporting); Writing‐review & editing (supporting).

## Supporting information

Fig S1

## Data Availability

The data sets analysed for this study were obtained from The Cancer Genome Atlas (TCGA) (https://portal.gdc.cancer.gov/), the International Cancer Genome Consortium (ICGC) (https://icgc.org/), the Gene Expression Omnibus (GEO) (https://www.ncbi.nlm.nih.gov/geo/) and UCSC Xena website (https://xenabrowser.net/).
