# Supplementary figures and images for: Development and validation of a CTNNB1‐associated metabolic prognostic model for hepatocellular carcinoma
Source: J Cell Mol Med. 2020 Dec 9;25(2):1151–65. doi: 10.1111/jcmm.16181 (PMC7812275; doi:10.1111/jcmm.16181)

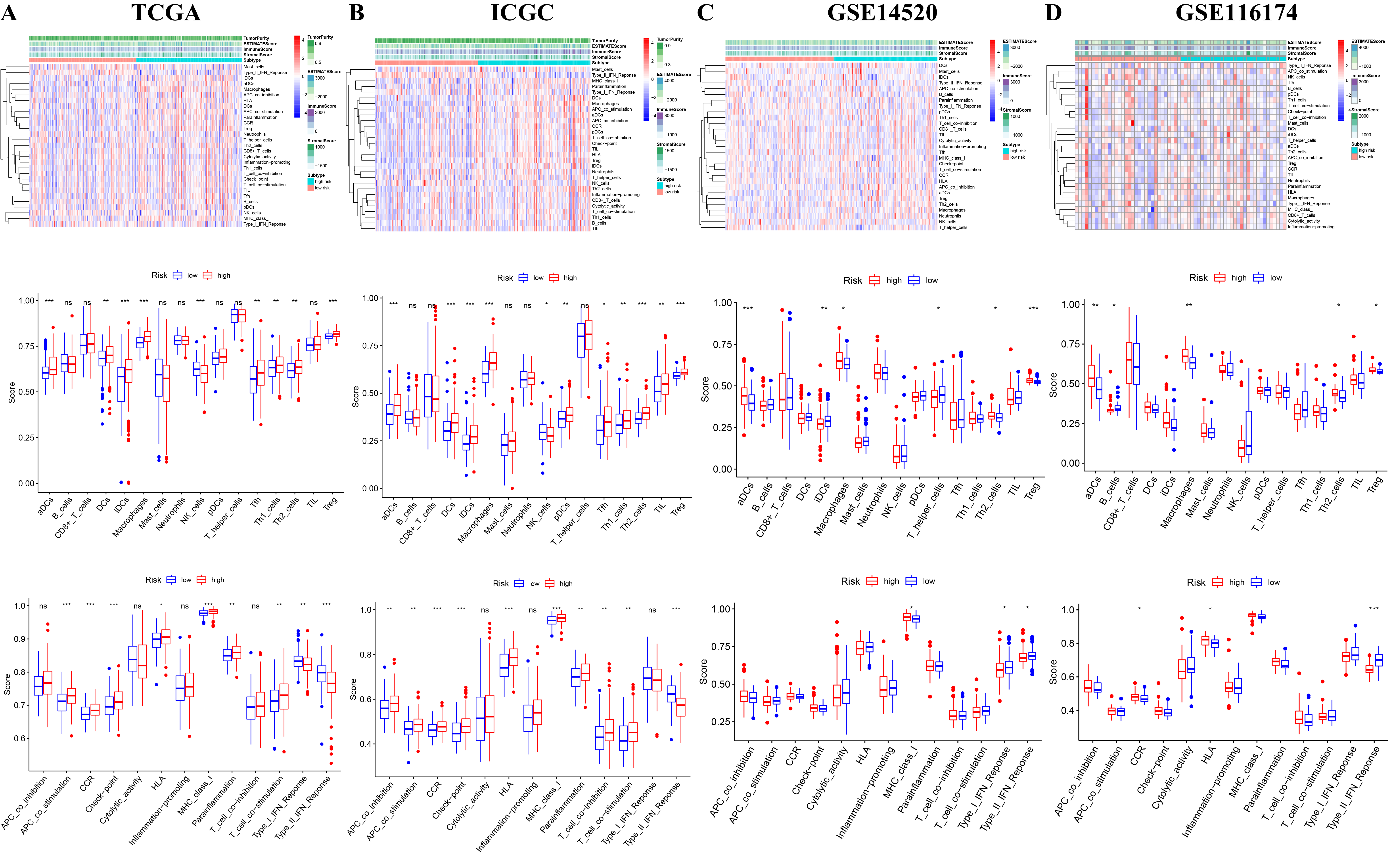

Supplement: Supplementary file 1 — Fig S1 [file JCMM-25-1151-s001.tif]
